# LncRNA LINC01512 Promotes the Progression and Enhances Oncogenic Ability of Lung Adenocarcinoma

**DOI:** 10.1002/jcb.26178

**Published:** 2017-06-22

**Authors:** Jie Chen, Fan Zhang, Junjun Wang, Lijuan Hu, Jian Chen, Gang Xu, Yumin Wang

**Affiliations:** ^1^ Department of Intensive Care Unit The First Affiliated Hospital of Wenzhou Medical University Wenzhou China; ^2^ Department of Laboratory Medicine The First Affiliated Hospital of Wenzhou Medical University Wenzhou China

**Keywords:** LUNG ADENOCARCINOMA, LONG NONCODING RNA, LINC01512, BIOLOGICAL FUNCTIONS

## Abstract

Previously, a significantly upregulated lncRNA, LINC01512, in lung adenocarcinoma (LAD) was obtained, while its biological function and molecular mechanisms were unclear. The expression level of LINC01512 was estimated by qPCR from 100 pairs of LAD and NT samples. The correlation of LINC01512 to clinical data of LAD patients was analyzed. LINC01512 was knocked down and overexpressed in SPCA‐1 and A549 cell lines by lentivirus‐mediated technology, and the oncological behavioral changes of SPCA‐1 and A549 cells were observed, as well as, tumorigenicity in experimental nude mice. Compared to the adjacent tissues, LINC01512 was obviously upregulated in LAD. The expression level of LINC01512 was closely related to lymph node metastasis and tumor node metastasis (TNM) stage. Survival analysis showed that the survival time of high expression LINC01512 group was significantly shorter than the low‐expression group in LAD. Knockdown or overexpression test unanimously confirmed that LINC01512 can increase the ability of cell migration, invasion, proliferation, colony formation, adhesion, and S phase and G2/M phase cells, whereas decrease the apoptosis and G0/G1 phase cells. Nude mice experiments confirmed that LINC01512 significantly enhanced the speed and weight of tumorigenicity. LINC01512 is an oncogenic lncRNA gene that promotes the progression and distinctly enhances the oncogenic ability in lung adenocarcinoma. J. Cell. Biochem. 118: 3102–3110, 2017. © 2017 The Authors. *Journal of Cellular Biochemistry* Published by Wiley Periodicals Inc.

Lung cancer is the leading cause of cancer‐related deaths worldwide with a continual increase in the number of incidences [Jemal et al., 2005]. Non‐small cell lung cancer (NSCLC) accounts for approximately 85% of all lung cancers. Histologically, NSCLC is divided into adenocarcinoma (LAD), squamous cell carcinoma (SCC), and large cell carcinoma. Although there has been some progress in chemotherapy, radiation, and surgery, lung cancer remains extremely aggressive leading to mortality [Gridelli et al., 2003]. The average 5‐year survival of lung cancer is less than 15% [Ogawa et al., 2008; Rachet et al., 2008; Chen et al., 2009; Stewart, 2010]. Recently, a growing proportion of LAD is a great concern for socioeconomic development and environment [Chen et al., 2016]. However, the mechanisms underlying LAD have not yet been elucidated.

Several studies have revealed that long non‐coding RNAs (lncRNAs) express irregularly in cancers and regulate the mRNA expression. The development, invasion, and metastasis in many cancers can be affected by the altered expression of lncRNAs as an effect of a combination of several mechanisms. [Fu et al., 2006; Gupta et al., 2010]. The gene expression was regulated by lncRNAs at different epigenetic, transcriptional, and post‐transcriptional levels [Chen et al., 2013; Hauptman and Glavac, 2013; Zhang et al., 2013]. LncRNAs have been shown to play critical roles in the development and progression of lung cancer. Since the degree of lung cancer correlates with few lncRNAs such as HOTAIR, H19, ANRIL, MALAT1 [Ji et al., 2003; Gibb et al., 2011], SCAL1 [Thai et al., 2013], AK126698 [Yang et al., 2013], and GAS6‐AS1 [Han et al., 2013], it is essential to determine the other lncRNAs and the underlying mechanisms.

Using high‐throughput microarray and qPCR, we found that lncRNA LINC01512 was upregulated in LAD. LINC01512, also known as LOC100132354, is localized at 6p21.1 with a total of 2282bp RNA sequence encompassing two exons. However, the clinical role and biological functions of LINC01512 are not well‐understood in LAD. In this study, the expression level of LINC01512 was estimated by qPCR in 100 pairs of LAD and NT samples, and the correlation of LINC01512 to clinical data in LAD patients was analyzed. Furthermore, we knocked down and overexpressed LINC01512 and analyzed the oncological behavioral changes of LAD cells.

## MATERIALS AND METHODS

### PATIENT SAMPLES

The study analyzed 100 LAD samples and corresponding NT samples collected from the First Affiliated Hospital of Wenzhou Medical University, China, from January 2013 to August 2015. The diagnosis of LAD was confirmed histopathologically, and the TNM clinical stages were determined based on the American Joint Committee on Cancer and the Union for International Cancer Control in 2002. The clinical data and characteristics of these patients are shown in Table I. These samples were preserved in liquid nitrogen postoperative. We monitored the rate of survival in these patients by telephone or other means and the longest follow‐up time was 22 months. The survival data were divided into the high‐ and low‐expression groups in the light of the expression level of LINC01512. All patients provided written informed consent, and the Institutional Ethics Review Board of the First Affiliated Hospital of Wenzhou Medical University approved this study.

**Table I:** The Clinical Features and the Relative Expression of LINC01512 of 100 Patients With LAD

	Cases (n)	LINC01512 relative expression^a^	Kruskal–Wallis test or Mann–Whitney *U*‐test	*P*
Sex			187.00	0.656
Male	40	0.630 (0.175–2.865)		
Female	60	1.034 (0.025–19.935)		
Clinical stage			10.325^b^	0.026
Ia	22	0.359 (0.095–1.297)		
Ib	31	0.612 (0.043–1.998)		
IIa	15	0.361 (0.098–11.962)		
IIb	16	1.194 (0.905–15.196)		
IIIa	15	11.202 (2.018–45.774)		
Differentiation degree			1.334^b^	0.890
Low	13	1.002 (0.164–14.415)		
Low‐moderate	14	1.021 (0.095–13.139)		
Moderate	36	0.466 (0.067–10.774)		
High‐moderate	19	0.735 (0.323–8.015)		
High	18	0.521 (0.098–7.276)		
LN metastasis			98.0	0.003
Yes	28	5.087 (0.954–88.452)		
No	72	0.512 (0.098–3.999)		
Smoking			279.00	0.089
Yes	37	0.734 (0.152–11.281)		
No	63	1.121 (0.110–18.021)		

^a^ The relative expression level of LINC01512 was used to calculate the relative lncRNA concentrations (ΔCt = Ct median lncRNA − Ct median β‐actin), and 2^−ΔΔCt^ was calculated as the relative expression.

^b^ Kruskal–Wallis test. LN: lymph node.

### QUANTITATIVE PCR

Total RNA was extracted from frozen LAD tissues and cell lines using TRIzol reagent (Invitrogen). According to the manufacturer's instructions, total RNA was reverse‐transcribed into cDNA using an RT Reagent Kit (Shanghai TaKaRa, China). LINC01512 and β‐actin mRNA expressions were measured by qPCR using SYBR Premix Ex Taq in ABI 7000 instrument. LINC01512 sense primer: 5′‐TTGGGCTGAGCACAGAGAAG‐3′, antisense primer: 5′‐GTAGGAGTGTGTGTGTGGGG‐3′; β‐actin sense primer: 5′‐CCTGGCACCCAGCACAAT‐3′, antisense primer: 5′‐GCTGATCCACATCTGCTGGAA‐3′. Two micrograms total RNA were transcribed to cDNA. PCR was performed in a total reaction volume of 20 µL, containing 10 µL SYBR Premix (2×), 2 µL cDNA template, 1 µL PCR forward primer (10 mM), 1 µL of PCR reverse primer (10 mM), and 6 µL double‐distilled water. The qPCR reaction included an initial denaturation step of 10 min at 95°C; 40 cycles of 5 s at 95°C, 30 s at 60°C; and a final extension step of 5 min at 72°C. All experiments were performed in triplicate, and all samples were normalized to β‐actin. The median in each triplicate was used to calculate the relative lncRNA concentrations (ΔCt = Ct median lncRNA − Ct median β‐actin), and 2^−ΔΔCt^ was calculated as the relative expression [Ren et al., 2012].

### CELL CULTURE

Five human LAD cell lines (SPCA‐1, NCI‐H1299, A549, NCI‐H441, and LTEP‐a2) and normal bronchial epithelial cells BEAS‐2B were purchased from the Cell Bank of the Chinese Academy of Sciences and cultured with complete medium (90% RPMI1640 or 90% DMEM supplemented with 10% fetal bovine serum; FBS) at 37°C, 5% CO_2_. The complete medium was replenished every 2–3 days.

### LENTIVIRUS‐MEDIATED siRNA, OVEREXPRESSION VECTOR CONSTRUCTION, AND TRANSFECTION

We constructed siRNA GV248 vector targeting LINC01512 and overexpression GV303 vector targeting LINC01512 (GeneChem, Shanghai, China). SiRNA sequences were as follows: siRNA1: CCACCTGGATAGGGACAAA, siRNA2: GAAGAGAAATTGACAGGTT, siRNA3: CTGGAGTAGAGAAGAGAAT. Transfections were performed by seeding 2 × 10^5^ cells in six‐well plates. After 24 h, the medium was replaced, and the cells incubated with the transfection complex according to the manufacturer's protocol; the multiplicity of infection (MOI) values were as follows: A549 MOI = 20 and SPCA‐1 MOI = 100. The cells were infected with lentivirus for 72 h, and the siRNA or overexpression efficiency was assessed by qPCR. Puromycin screen test isolated the cell lines successfully transfected with the lentivirus‐mediated vector. The study included SPCA‐1 LINC01512 siRNA cell lines (including siRNA1, siRNA2, siRNA3 groups), SPCA‐1 was infected with lentivirus negative control LVCON077 vector (NC group), SPCA‐1 (control group), and A549 LINC01512 overexpressing cell line (LINC01512 group) and A549 was infected with lentivirus negative control LVCON145 vector (NC group), and A549 (control group).

### CELL PROLIFERATION ASSAY

Cell proliferation was evaluated by Cell Counting Kit‐8 (CCK‐8, Corning Corporation) according to the manufacturer's protocols. Briefly, 3,000 cells were resuspended and seeded in a 96‐well plates in the presence of 10% FBS and cultured for 1 week. The next day, the cells were incubated with 10 μL CCK‐8 for 1 h, and the absorbance was measured at 450 nm with a multifunctional microplate reader (Tecan, German) on 1, 3, 5, and 7 days.

### CELL MIGRATION ASSAY

Three vertical lines were marked on the back of six‐well plates, three different A549 cell groups (about 5 × 10^5^/well) added, washed three times with PBS, and serum‐free medium added before imaging at 0 h. The plates were placed in the cell incubator, and changes analyzed after 24 h.

### CELL INVASION ASSAY

Invasion assay was performed with 8.0‐µm pore inserts (Millipore) in a 24‐well plates. The invasion assay was performed with Matrigel‐coated filters (Sigma Corporation). Cells were allowed to incubate for 24 h and 48 h, respectively. Migrated and invaded cells were fixed with methanol and stained with 0.1% (w/v) crystal violet, followed by bleaching with 33% acetic acid and measured at 570 nm on a microplate reader.

### ADHESION ASSAY

The 96‐well plates was processed with 50 µL Matrigel and fibronectin (FN; 50 μg/mL), and no processed wells were categorized as CON group. 2 × 10^4^ cells/well were added and stained using 0.1% (w/v) crystal violet, quenched using 2% SDS, and measured at 550 nm.

### APOPTOSIS DETECTION

The activities of caspase‐3 and caspase‐9 were detected by spectrophotometry. The caspase family plays a vital role in mediating the process of apoptosis. The caspase‐3 and caspase‐9 are critical execution molecules and inactive under normal circumstances; they are active as apoptotic molecules. The absorbance at 405 nm may indicate the degree of activation of caspases.

### CELL CYCLE ASSAY

The cells were harvested by centrifugation and fixed using 70% ethanol at 4°C overnight. The cells were resuspended in 400 µL PBS (containing 2 mg/mL RNA enzyme) and incubated at 37°C for 30 min. Four‐hundred microliters of propidium iodide (0.1 mg/mL) were incubated for 10 min and DNA content detected by flow cytometry (Cytomics FS‐500, Beckman Coulter). The results were analyzed using MultiCycle software.

### COLONY FORMATION ASSAY

The cells in six‐well plates were digested after cultivating for 48 h, then adjusted for the number of cells (300 cells), and 50 µL was added to six‐well plates, mixed, and incubated at 37°C for 14 days. The resulting colonies were fixed with glutaraldehyde (6.0% v/v), stained with crystal violet (0.5% w/v), and counted using a stereomicroscope. Subsequently, more than 50 cells counted were cloned for the calculation: colony formation rate (%) = (number of colonies/number of inoculated cells) × 100%.

### NUDE MICE TUMOR FORMATION

The cells include SPCA‐1 LINC01512 siRNA2 cell line (siRNA2 group), lentivirus negative control LVCON077 was infected with SPCA‐1 (NC group). A549 LINC01512 overexpressing cell line (LINC01512 group) and lentivirus negative control LVCON145 were infected with A549 (NC group). The axillary subcutaneous of BALB/c nude mice was inoculated with 2 × 10^6^ cells from four groups, followed by the measurement of the body weight and tumor size. Finally, the mice were sacrificed and frozen in liquid nitrogen.

### STATISTICAL METHODS

Differences in variables between the groups were evaluated using one‐way ANOVA for normal distribution or Kruskal–Wallis test for the non‐normal distribution. A comparison between the two groups was performed by least significant difference (LSD) test or Student's *t*‐test or Mann–Whitney *U*‐test. Survival curve was drawn using GraphPad Prism 6 software, and chi‐square test analyzed the differences. *P *< 0.05 was considered to be statistically significant.

## RESULTS

### THE EXPRESSION LEVEL OF LINC01512 IN LAD AND ADJACENT TISSUES AND CORRELATION WITH CLINICAL DATA

**Figure 1:**
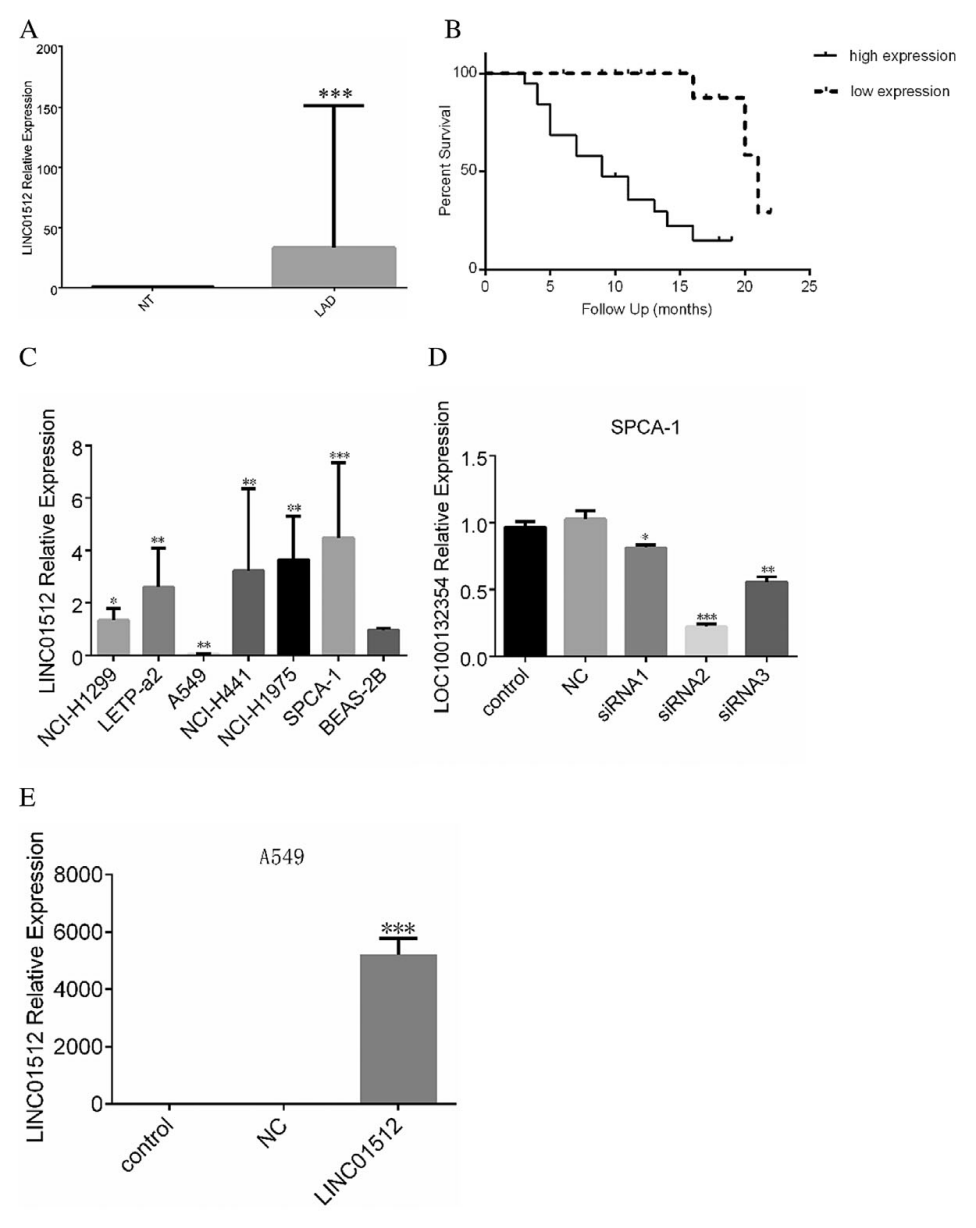
Expression level of LINC01512 in LAD tissue, cells, three A549 cell groups, three SPCA‐1 cell groups. (A) Comparison of the relative expression levels of LINC01512 in LAD and adjacent tissues. LINC01512 expression level of LAD was significantly higher than the adjacent tissue. **P < 0.01. (B) The correlation of LINC01512 expression with the survival of LAD prognosis. The survival time of LINC01512 high‐expression group was significantly shorter than that of the low‐expression group. (C) Comparison of LINC01512 expression levels in six LAD cell lines. The expression levels of LINC01512 from NCI‐H1975, SPCA‐1, and NCI‐H441 cells were highly expressed. The expression level of SPCA‐1 was the highest, and that of NCI‐H1299 and LETP‐a2 was moderate, while that of A549 cells was the lowest. *P < 0.05, **P < 0.01, ***P < 0.001. (D) Comparison of interference efficiency of PCR amplification of three LINC01512 interference sequences. siRNA2 group exhibited the highest interference efficiency. (E) Overexpression of LINC01512 in three A549 cell groups. The overexpression level of LINC01512 in A549 cell line was significantly higher than that in the control and NC groups. *P < 0.05, **P < 0.01, ***P < 0.001. The relative expression level of LINC01512 was used to calculate the relative lncRNA concentrations (ΔCt = Ct median lncRNA − Ct median β‐actin), and 2^−ΔΔCt^ was calculated as the relative expression.

According to Table I and Figure 1A, the relative expression level of LINC01512 in LAD tissues is 8.217 (0.120–90.235) and significantly higher than the corresponding adjacent cancer tissues (Mann–Whitney *U* = 103.00, *P* = 0.004). We showed that the LINC01512 level of LAD with lymph node metastasis group was significantly higher than that of LAD without the lymph node metastasis group (Mann–Whitney *U* = 98.000, *P* = 0.003). The expression level of LINC01512 in different TMN stages was different (Kruskal–Wallis test = 10.325, *P* = 0.026), and the expression level of LINC01512 in stage IIIa was significantly higher than that in stages Ia (Mann–Whitney *U* = 35.000, *P* < 0.001), Ib (Mann–Whitney *U* = 15.000, *P* = 0.008), IIa (Mann–Whitney *U* = 15.000, *P* = 0.026), and IIb (Mann–Whitney *U* = 15.000, *P* = 0.023). The expression of LINC01512 did not associate with the histological differentiation (Kruskal–Wallis test = 1.334, *P* = 0.890), smoking (Mann–Whitney *U* = 279.00, *P* = 0.089), sex (Mann–Whitney *U* = 187.00, *P* = 0.656), and age (Pearson = −0.079, *P* = 0.780). According to Figure 1B, the LINC01512 high‐expression group had a significantly shorter survival (median 9.2 months) than the low‐expression group (median 21.4 months) (χ^2^ = 16.26, *P *< 0.001).

### EXPRESSION LEVEL OF LINC01512 FROM FIVE LAD CELLS

Compared to the normal human bronchial epithelial BEAS‐2B cell line, we assessed the expression levels of LINC01512 from five LAD cell lines (A549, NCI‐H441, NCI‐H1299, SPCA‐1, LETP‐a2) by qPCR. The expression levels of LINC01512 in NCI‐H1975, SPCA‐1, and NCI‐H441 cells were high (*P* = 0.0022, 0.000, 0.0032) and that of NCI‐H1299 and LETP‐a2 was moderate, while that of A549 cells was lowest (Fig. 1C).

### DETERMINATION OF THE OPTIMAL INTERFERENCE SEQUENCE FOR SPCA‐1 CELL LINE, LINC01512, AND OVEREXPRESSION LEVEL IN A549 CELLS

As shown in Figure 1D, siRNA1, siRNA2, and siRNA3 were interfered with control and NC groups (*P* = 0.032, 0.000, 0.006). Thus, the interference efficiency of siRNA2 was the highest rendering it as the optimal interference sequence. The overexpression level of LINC01512 in LINC01512 group (4876.34 ± 913.45) was significantly higher than that in the control and NC groups (*P* = 0.000; Fig. 1E).

### LINC01512 WAS RELATIVE TO CELL MIGRATION AND INVASION

**Figure 2:**
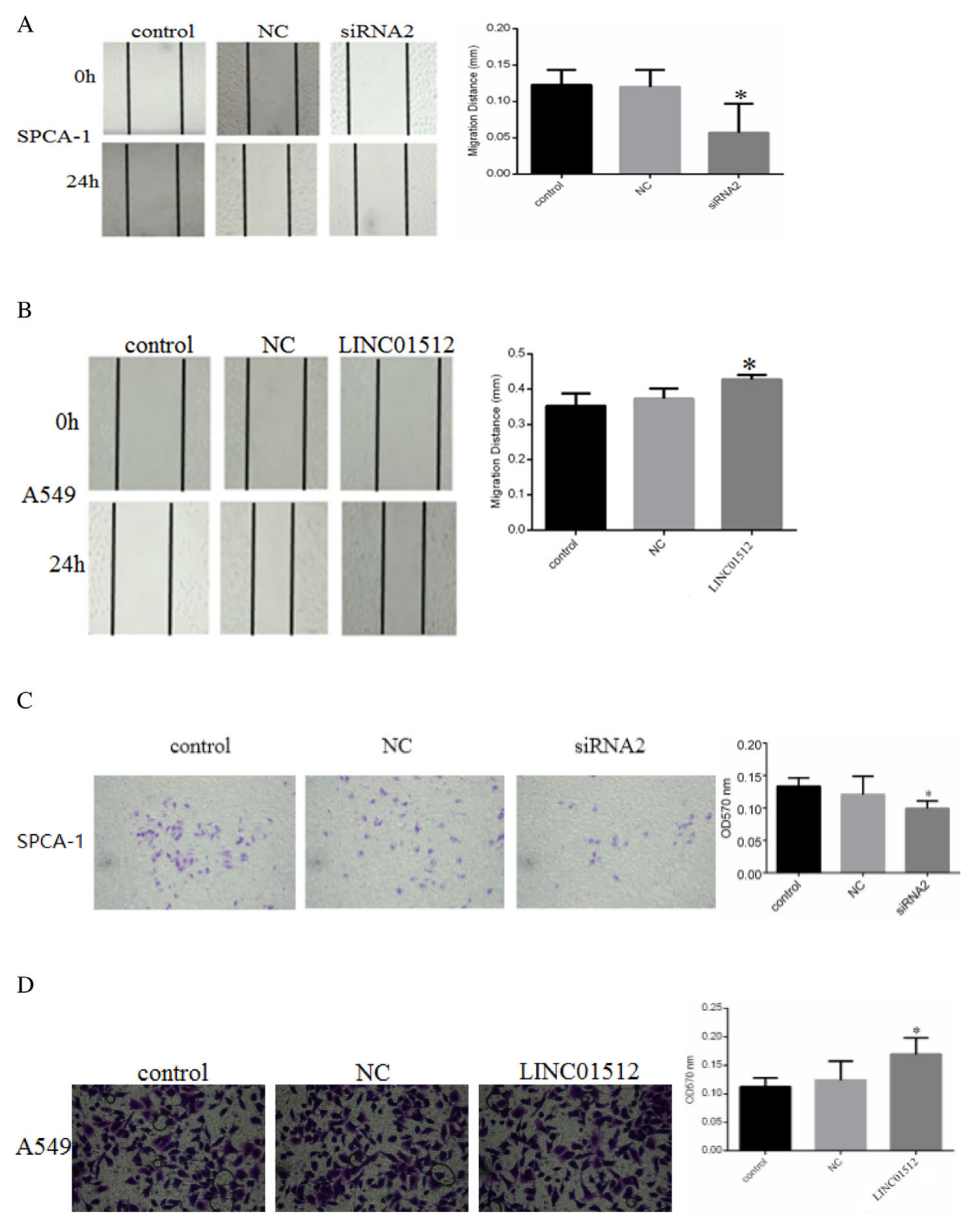
LINC01512 is relative to ability of cell migration and invasion in LAD. (A) Results of cell migration of SPCA‐1 cells. Compared to the NC and control groups, the migration distance of siRNA2 group decreased significantly after LINC01512 was knocked down. (B) Results of cell migration of A549 Cells. Compared to the NC and control groups, the migration distance of LINC01512 group increased significantly after LINC01512 was overexpressed. (C) Comparison of the results of invasion test in different treatment SPCA‐1 cells groups. The OD570 nm of siRNA2 group decreased significantly after LINC01512 was knocked down. (D) Comparison of the results of cell invasion test in different treatment A549 cells groups. The OD570 nm of LINC01512 group increased significantly after LINC01512 was overexpressed. *P < 0.05.

After LINC01512 was knocked down, compared to the NC group (0.138 ± 0.029 mm) and control group (0.141 ± 0.021 mm), the migration distance of siRNA2 group (0.064mm ± 0.015 mm) decreased significantly (*P* = 0.021, 0.019) (Fig. 2A). The cell invasion test showed that the 570 nm absorbance (OD570 nm) of siRNA2 group was lower than control and NC groups (Fig. 2C, *P* = 0.017, 0.021). After LINC01512 was overexpressed, compared to the control (0.353mm ± 0.036 mm) and NC groups (0.380mm ± 0.034 mm), the cell migration distance of LINC01512 group (0.454mm ± 0.017 mm) increased significantly (Fig. 2B, *P* = 0.033, 0.029). The cell invasion test showed that OD570 of LINC01512 group was higher than the control and NC groups (Fig. 2D, *P* = 0.023, 0.028).

### LINC01512 PROMOTED CELL PROLIFERATION AND INHIBITED CELL APOPTOSIS

**Figure 3:**
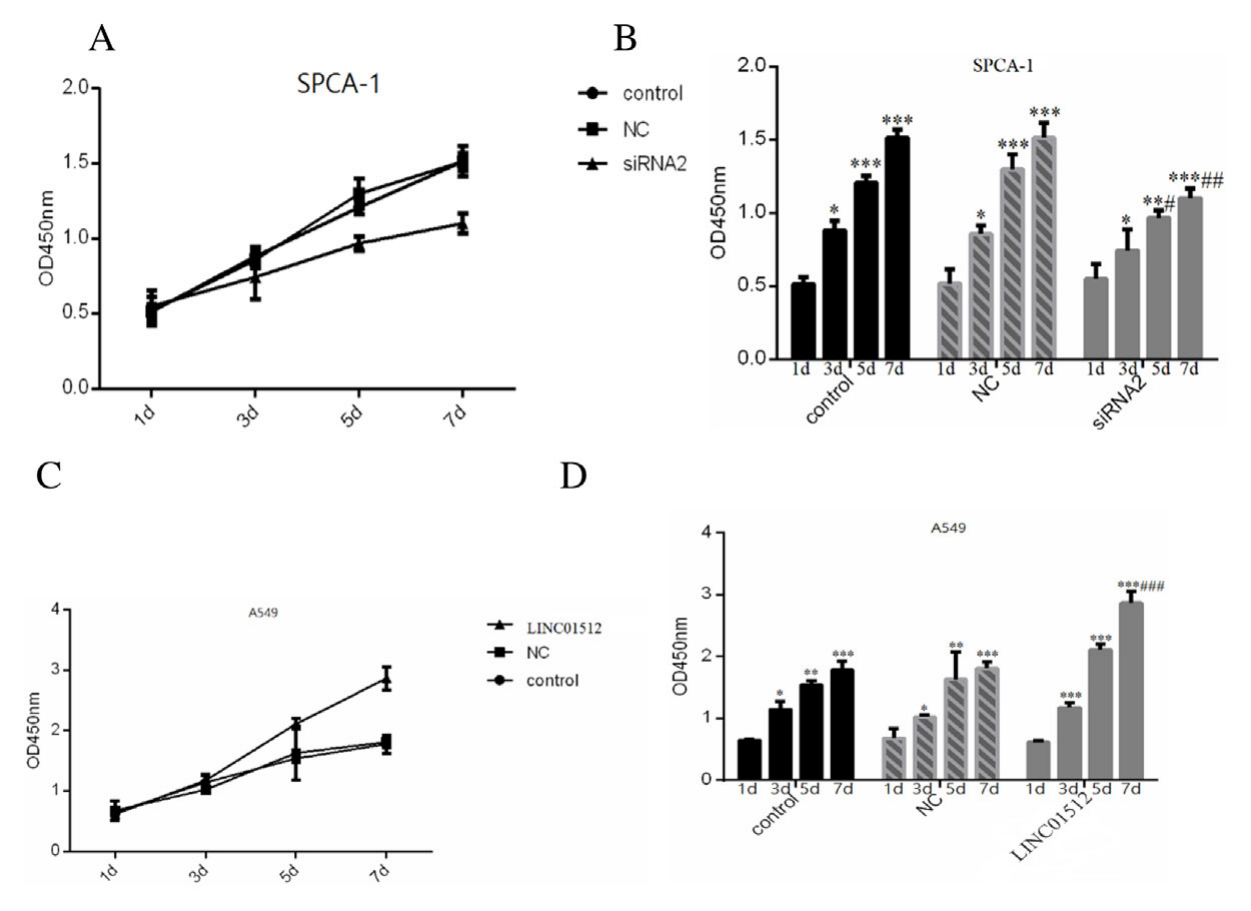
Comparison of cell proliferation in three different treatment groups on different days of the CCK‐8 assay. (A) The curve of three SPCA‐1 groups of different days. (B) Illustration of three SPCA‐1 groups of different days. (C) The curve of three A549 groups of different days. (D) The illustration of three A549 groups of different days. *P < 0.05, **P < 0.01, ***P < 0.001, #*P* < 0.05, ##*P* < 0.01.

**Figure 4:**
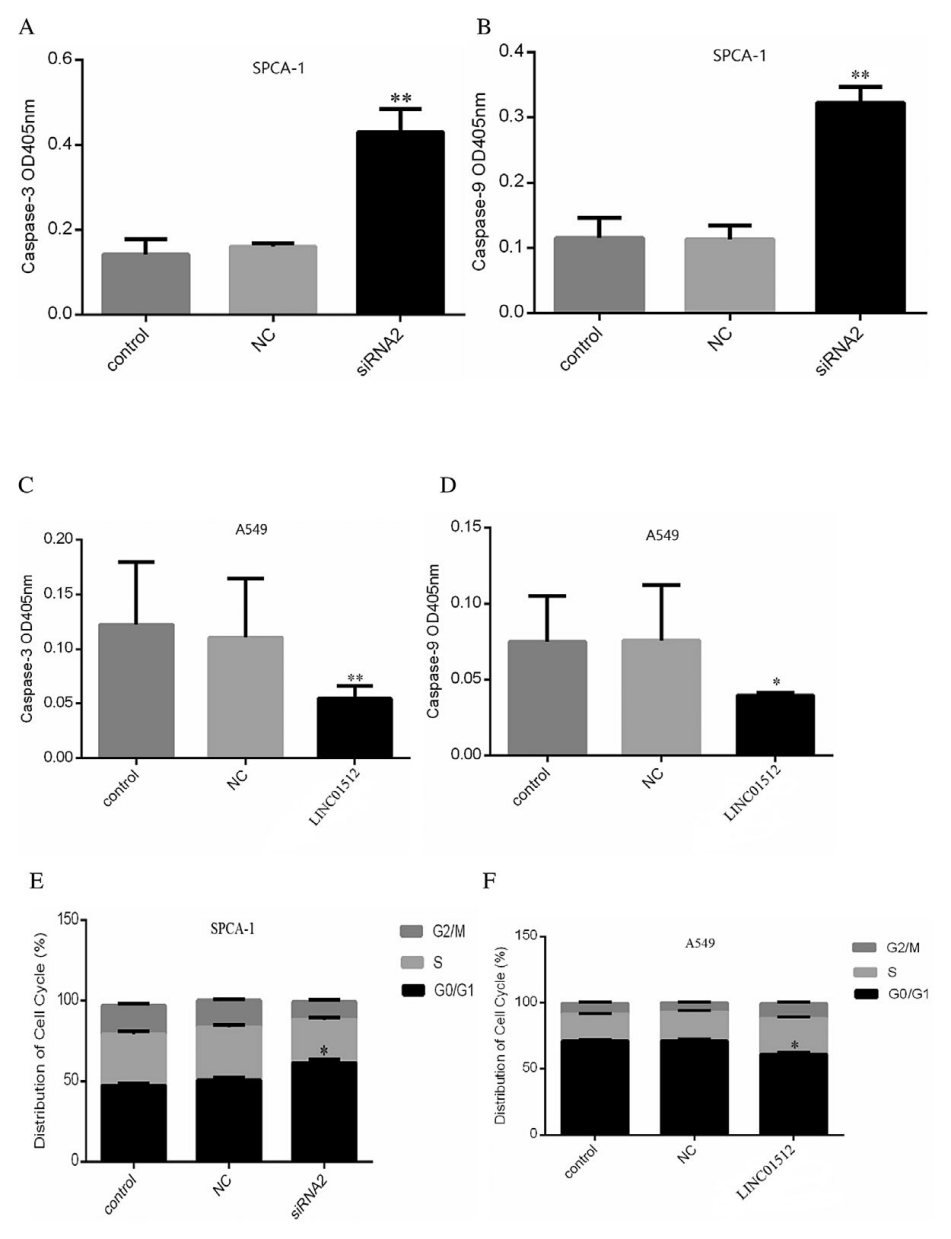
Results of cell apoptosis and cell cycles. (A) Activity of caspase‐3 changes in the different SPCA‐1 group. (B) Activity of caspase‐9 changes in different SPCA‐1 groups. (C) Activity of caspase‐3 changes in different A549 groups. (D) Activity of caspase‐9 changes in different A549 groups. (E) The result of cell cycle in the control of the SPCA‐1 group. (F) Result of cell cycles in control of A549 group. *P < 0.05, **P < 0.01.

In Fig. 3A and B, OD450 of different SPCA‐1 groups gradually increased with duration. Compared to 1 day, the OD450 of control, NC, and siRNA2 groups from SPCA‐1 cells in 3 (*P* < 0.05, *P* < 0.05, *P* < 0.05), 5 (*P* < 0.01, *P* < 0.001, *P* < 0.001), and 7 days (*P* < 0.001, *P* < 0.001, *P* < 0.001) increased significantly. Compared to the appropriate days of control and NC groups, the OD450 of 1 day and 3 days in the siRNA2 group was not significantly different (*P *> 0.05), while that of 5 days (*P* < 0.05) and 7 days (*P* < 0.01) were significantly reduced. Similarly, after LINC01512 was overexpressed (Fig. 3C and D), and OD450 of different A549 groups increased gradually with duration. Compared to 1 day, the OD450 of 3 days (*P *< 0.001), 5 days (*P* < 0.001), 7 days (*P* < 0.001) in the LINC01512 group increased significantly. No significant difference was observed at OD450 of LOC100132345 group between the control and NC groups (F = 0.348, *P *= 0.720; F = 2.298, *P* = 0.182; F = 3.946, *P* = 0.081) on 1 day, 3 days, and 5 days, while it was significantly higher than the control and NC groups (F = 50.385, *P* <0.001; *P* <0.001, <0.001) on 7 days. Apoptosis assay showed that the activities of caspase‐3 and caspase‐9 in the siRNA2 group were significantly higher than those in the control and NC groups (*P* = 0.0021, 0.0013 and *P* = 0.0035, 0.0041) as shown in Figure 4A and B. SPCA‐1 cell apoptosis was significantly increased after LINC01512 was knocked down. Figure 4C and D showed that the activities of caspase‐3 and caspase‐9 in the LINC01512 group were significantly lower than those in the control and NC groups (*P* = 0.0017, 0.0029 and 0.027, 0.019); thus, LINC01512 exerted an inhibitory effect on cell apoptosis. As shown in Figure 4E, the the cells of G0/G1 phase in the SPCA‐1 siRNA2 group were significantly increased as compared to the control and NC groups, while those in the S and G2/M phase were decreased significantly (*P *= 0.012, 0.018). Similarly, in Figure 4F, after LINC01512 was overexpressed, S phase (*P* < 0.001 and *P* < 0.001) and G2/M phase cells (*P* < 0.001 and *P* < 0.001) of the LINC01512 group were reduced significantly, whereas apoptosis and G0/G1 phase cells (*P *< 0.001 and *P *< 0.001) were increased remarkably than the control and NC groups. The cell cycle result further confirmed that LINC01512 increased the ability of LAD cell proliferation.

### LINC01512 SIGNIFICANTLY INCREASED THE CELL ADHESION OF LAD

**Figure 5:**
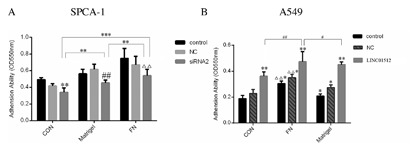
Comparison of the results of adhesion. (A) Adhesion of three SPCA‐1 cell groups; compared to the CON group of siRNA2 groups, the OD550 of Matrigel and FN groups of siRNA2 groups significantly increased after Matrigel and FN treatment. The OD550 in the siRNA2 groups was lower than that in the control and NC groups (*P* = 0.0044, 0.0038, 0.0032) after CON, Matrigel, and FN treatment, indicating that LINC01512 siRNA resulted in significantly reduced cell adhesion. Compared to the Matrigel and CON groups, NC and siRNA2 groups were significantly higher than the control groups after FN treatment. (B) Adhesion of three A549 cell groups. Compared to the CON group, OD550 in LINC01512 groups was significantly higher than that in the NC and control groups (*P* = 0.0012, 0.0041, *P* = 0.0059, 0.0093) after Matrigel and FN treatment. The OD550 of control, NC, and LINC01512 groups was significantly higher than the Matrigel and CON groups (*P* = 0.033, 0.026, 0.011 and *P* = 0.0076, 0.0042, 0.0018) after FN treatment. *P < 0.05, **P < 0.01, ***P < 0.001, #*P* <0.05, ##*P* < 0.01, △*P* < 0.05, △△*P* < 0.01.

The 96‐well plates were coated with FN and Matrigel, and the uncoated wells were used as controls (CON). Compared to the CON group of siRNA2 groups, the OD550 of siRNA2 groups significantly increased after Matrigel and FN treatment. The OD550 in siRNA2 groups was lower than that in the control and NC groups (*P* = 0.0044, 0.0038, 0.0032) after Matrigel and FN treatment (Fig. 5A), indicating that LINC01512 siRNA decreased the cell adhesion significantly. Compared to the Matrigel and CON groups, the NC and siRNA2 groups were significantly higher than the control groups (*P* < 0.01, *P* < 0.01) after FN treatment. Similarly, compared to the CON group, OD550 in LINC01512 groups was significantly higher than that in the NC and control groups (*P* = 0.0012, 0.0041, *P* = 0.0059, 0.0093) after Matrigel and FN treatment. The OD550 of control, NC, and LINC01512 groups was significantly higher than the Matrigel and CON groups (*P* = 0.033, 0.026, 0.011 and *P* = 0.0076, 0.0042, 0.0018) after FN treatment (Fig. 5B). The experiment confirmed that LINC01512 significantly increased the cell adhesion of LAD.

### LINC01512 UNCOMMONLY ADVANCED THE CELL CLONE FORMATION OF LAD

**Figure 6:**
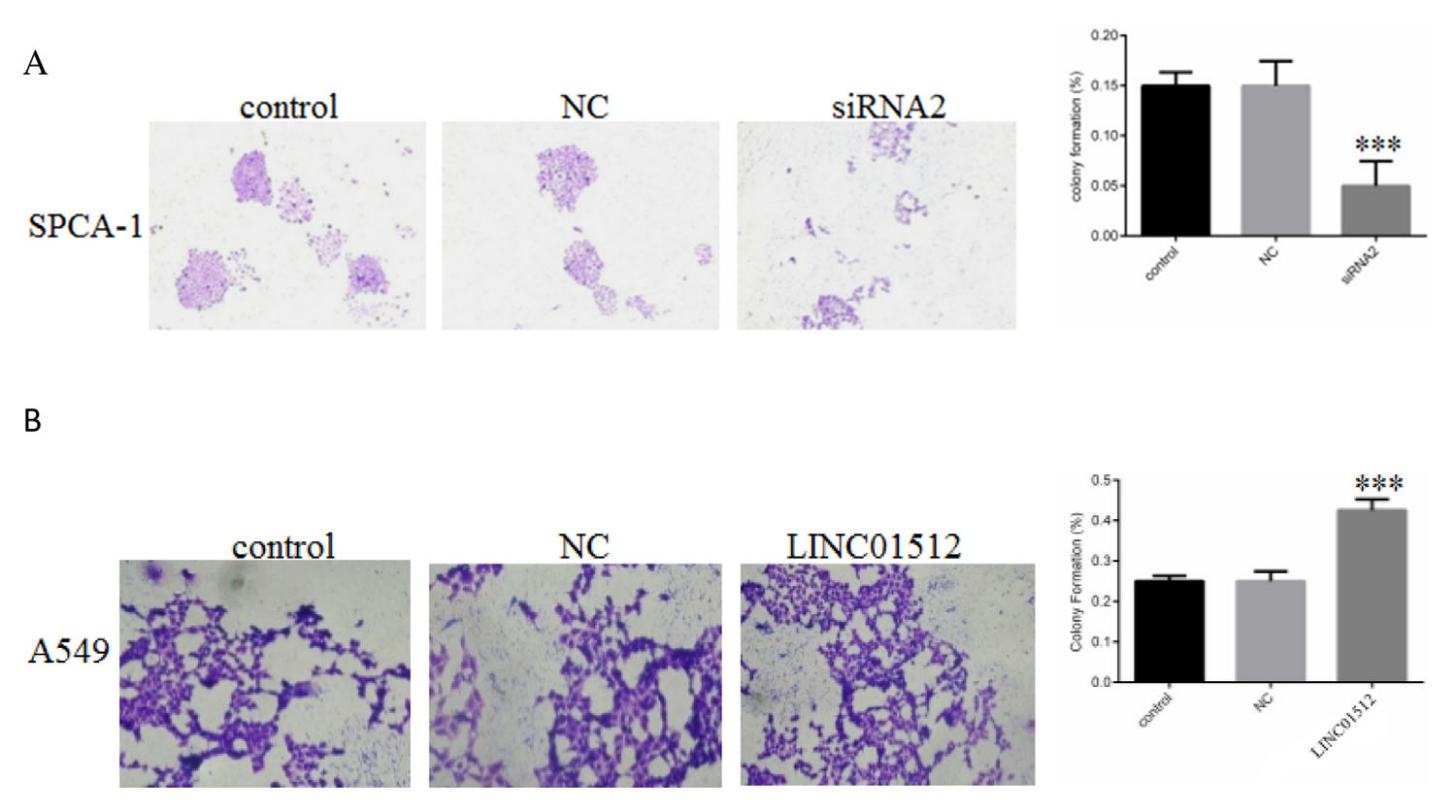
Results of cell clone formation were compared. (A) The clone formation rate of siRNA2 group (5.00 ± 0.81%) was significantly lower than that of the control (14.97 ± 1.34%, *P* = 0.0018) and NC groups (15.13 ± 2.31%, *P* = 0.0011). (B) Clone formation rate in the control (25.01 ± 1.37%, *P* < 0.001) and NC groups (26.15 ± 2.48%, *P* < 0.001) was significantly lower than that in the LINC01512 group (42.57 ± 2.71%) after LINC01512 overexpression. **P < 0.01, ***P < 0.001.

The rate of clone formation of the siRNA2 group (5.00 ± 0.81%) was significantly lower than that of the control group (14.97 ± 1.34%, *P* = 0.0018) and NC group (15.13 ± 2.31%, *P* = 0.0011) (Fig. 6A). The rate of clone formation in the control group (25.01 ± 1.37%, *P* < 0.001) and NC group (26.15 ± 2.48%, *P* < 0.001) was significantly lower than that in the LINC01512 group (42.57 ± 2.71%) after LINC01512 overexpression (Fig. 6B). Thus, LINC01512 was observed to uncommonly advance the cell clone formation of LAD.

### LINC01512 SIGNIFICANTLY ENHANCED THE TUMORIGENIC ABILITY OF THE NUDE MICE

**Figure 7:**
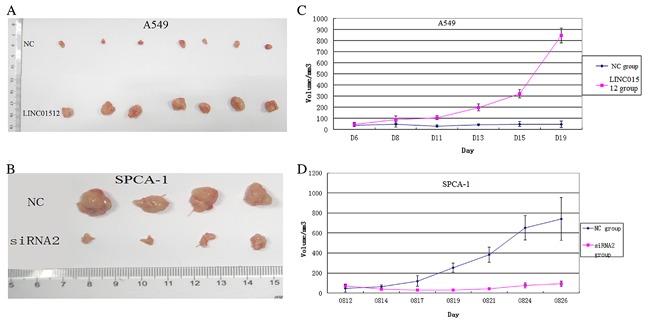
Experimental results of nude mice. (A) Tumor volume of LINC01512 and NC groups of A549 cells. The rate of tumor growth of the LINC01512 group was significantly faster than that of the NC group (*P* < 0.05). (B) Tumor volume of siRNA2 and NC of SPCA‐1 cells. The rate of tumor growth of the siRNA2 group was significantly slower than that of the NC group (*P* < 0.05). (C) The growth curve of the tumor from LINC01512 and NC groups of A549 cells. The tumor weight in the LINC01512 group was significantly higher than that in the NC group (t = 4.831, *P* < 0.001). (D) The growth curve of the tumor from siRNA2 and NC groups of SPCA‐1 cells. The tumor weight in the siRNA2 group was significantly lower than that in the NC group (t = 6.327, *P* < 0.001).

The rate of tumor growth of the LINC01512 group was significantly accelerated than that of the NC group (*P* < 0.05) (Fig. 7A). The tumor weight in the LINC01512 group was significantly higher than that in the NC group (t = 4.831, *P* < 0.001) (Fig. 7C). On the other hand, the rate of tumor growth of the siRNA2 group was significantly slower than that of the NC group (*P* < 0.05) (Fig. 7B). The tumor weight in the siRNA2 group was significantly lower than that in the NC group (t = 6.327, *P *< 0.001) (Fig. 7D). Thus, LINC01512 significantly enhanced the tumorigenic ability of the nude mice.

## DISCUSSION

LncRNAs are involved in several physiological processes, such as X chromosome inactivation and gene imprinting [Drobnik et al., 1999; Wang and Chang, 2011]. In addition, gene expression is regulated along with the development and progression of tumors [Gupta et al., 2010; Khachane and Harrison, 2010]. Promoters can bind some transcription factors (TFs) by a series of mechanisms as chromosomal rearrangements and transfer accessories [Loh et al., 2006]. The activity of lncRNAs can regulate the expression of nearby encoding genes by affecting the process of transcription [Mattick and Gagen, 2001] or directly playing an enhancer‐like role [Mattick, 2010; Orom et al., 2010].

The lentiviral vector can transfect the target cell line easily and efficiently than the common vector, which is a simple and convenient procedure. The cell line can be screened by the antibiotic marker gene or the green fluorescent protein gene as *EGFP* (or GFP).

In the present study, we discovered the potential role of LINC01512 in the pathogenesis of LAD. qPCR revealed that LINC01512 overexpressed and closely related to lymph node metastasis and TNM stage in LAD patients. Survival analysis showed that the survival time of highly expressing LINC01512 patients was shorter than that of low LINC01512 level from patients in LAD, thereby suggesting that LINC01512 is a potential biomarker and an intervention target in LAD.

Furthermore, knockdown or overexpression assay confirmed that LINC01512 can increase the ability of cell migration, invasion, proliferation, colony formation, adhesion, and S phase, G2/M phase cells, whereas decrease apoptosis and G0/G1 phase cells. Nude mice experiment confirmed that LINC01512 significantly enhanced the speed and weight of tumorigenicity, indicating that LINC01512 can enhance the rate of proliferation, ability of cell colony formation, and adhesion while inhibiting cell apoptosis in LAD cells.

Our study ascertained that LINC01512 enhanced the ability of cell migration, invasion, proliferation, colony formation, adhesion, and S phase, G2/M phase cells, while decrease the apoptosis and G0/G1 phase cells. The molecule significantly enhanced the ability of tumorigenicity of nude mice. Our results confirmed that the LINC01512 gene is a oncogenic lncRNA gene and a potential biomarker of LAD, and therefore, a new therapeutic target.
